# Prion protein (PrP) gene-knockout cell lines: insight into functions of the PrP

**DOI:** 10.3389/fcell.2014.00075

**Published:** 2015-01-15

**Authors:** Akikazu Sakudo, Takashi Onodera

**Affiliations:** ^1^Laboratory of Biometabolic Chemistry, Faculty of Medicine, School of Health Sciences, University of the RyukyusNishihara, Japan; ^2^Research Center for Food Safety, School of Agricultural and Life Sciences, University of TokyoTokyo, Japan

**Keywords:** prion protein, *Prnp*^−/−^ cell line, Doppel, Shadoo, knockout mouse

## Abstract

Elucidation of prion protein (PrP) functions is crucial to fully understand prion diseases. A major approach to studying PrP functions is the use of PrP gene-knockout (*Prnp*^−/−^) mice. So far, six types of *Prnp*^−/−^ mice have been generated, demonstrating the promiscuous functions of PrP. Recently, other PrP family members, such as Doppel and Shadoo, have been found. However, information obtained from comparative studies of structural and functional analyses of these PrP family proteins do not fully reveal PrP functions. Recently, varieties of *Prnp*^−/−^ cell lines established from *Prnp*^−/−^ mice have contributed to the analysis of PrP functions. In this mini-review, we focus on *Prnp*^−/−^ cell lines and summarize currently available *Prnp*^−/−^ cell lines and their characterizations. In addition, we introduce the recent advances in the methodology of cell line generation with knockout or knockdown of the PrP gene. We also discuss how these cell lines have provided valuable insights into PrP functions and show future perspectives.

## Introduction

Prion diseases, also called transmissible spongiform encephalopathies (TSE), are a group of neurodegenerative disorders that affect both humans and animals (Prusiner, 1998). Examples of these disorders in humans include Creutzfeldt-Jakob disease (CJD), Gerstmann-Sträussler-Scheinker syndrome (GSS), kuru and fatal familial insomnia (FFI). TSEs found in animals include scrapie, bovine spongiform encephalopathy (BSE) and chronic wasting disease (CWD). The representative characteristics of prion diseases are spongiform changes, which are associated with neuronal loss, vacuolation, astrocytosis, formation of amyloid plaques and a failure to induce an inflammatory response (Sakudo and Ikuta, 2009a,b). A common characteristic of prion diseases is rapid symptom progression after a prolonged incubation period. The damaged region of the brain differs among each prion disease and determines the clinical signs and symptoms of the specific disease. Although the incidence of prion diseases is rare, it is fatal because of a lack of effective treatment to either cure or delay disease progression. Furthermore, the risk of prion transmission is a serious threat to public health due to the high resistance of prion agents to standard sterilization techniques (Sakudo et al., 2011a).

Prion protein (PrP) is the most important factor for prion infection. This finding has been verified by experiments using PrP gene (*Prnp*)-knockout (*Prnp*^−/−^) mice and cells derived from these mice (Sakudo et al., 2006, 2007a,b). The first important finding was that *Prnp*^−/−^ mice are resistant to infection with prion agents (Bueler et al., 1993; Prusiner et al., 1993; Manson et al., 1994a,b). Secondly, *Prnp*^−/−^ primary culture neurons are not killed by the toxicity of prion agents or PrP(106-126) (Brandner et al., 1996a; Giese et al., 1998). Thirdly, prions cannot proliferate in the brain of *Prnp*^−/−^ mice (Brandner et al., 1996b). Therefore, it is apparent that PrP plays an important role in the mechanisms of infection and contributes to the pathogenesis of prion diseases. This is also consistent with the notion that the conversion of cellular PrP (PrP^C^) into abnormal PrP (PrP^Sc^) constitutes a fundamental feature of prion diseases (Prusiner, 1998). It seems that PrP^C^ acts as a cellular receptor of PrP^Sc^. Because PrP^C^ is thought to function as a dimeric form (Meyer et al., 2000; Kaimann et al., 2008), PrP^C^-PrP^Sc^ interaction might alternate between PrP^C^-PrP^Sc^ and PrP^Sc^-PrP^Sc^ after prion infection. As shown above, *Prnp*^−/−^ mice and the derived cells have confirmed the importance of PrP in the pathogenicity of prion diseases and have greatly contributed to the understanding of these disorders.

However, despite the numerous studies on *Prnp*^−/−^ mice, the biological functions of PrP^C^, which are crucial for understanding prion diseases, remain elusive. Cell lines are useful for detailed analysis of gene function. Therefore, to investigate PrP^C^ functions in detail, *Prnp*^−/−^ cell lines derived from *Prnp*^−/−^ mice have been established. The present review will summarize currently available *Prnp*^−/−^ cell lines. In addition, recent advances in strategies for producing cell lines in which *Prnp* is knocked out or knocked down will be presented. Valuable insights into PrP^C^ functions made possible by the availability of these cell lines will be introduced as well.

## The analysis of *Prnp^−/−^* mice for understanding PrP^C^ function

**Figure 1:**
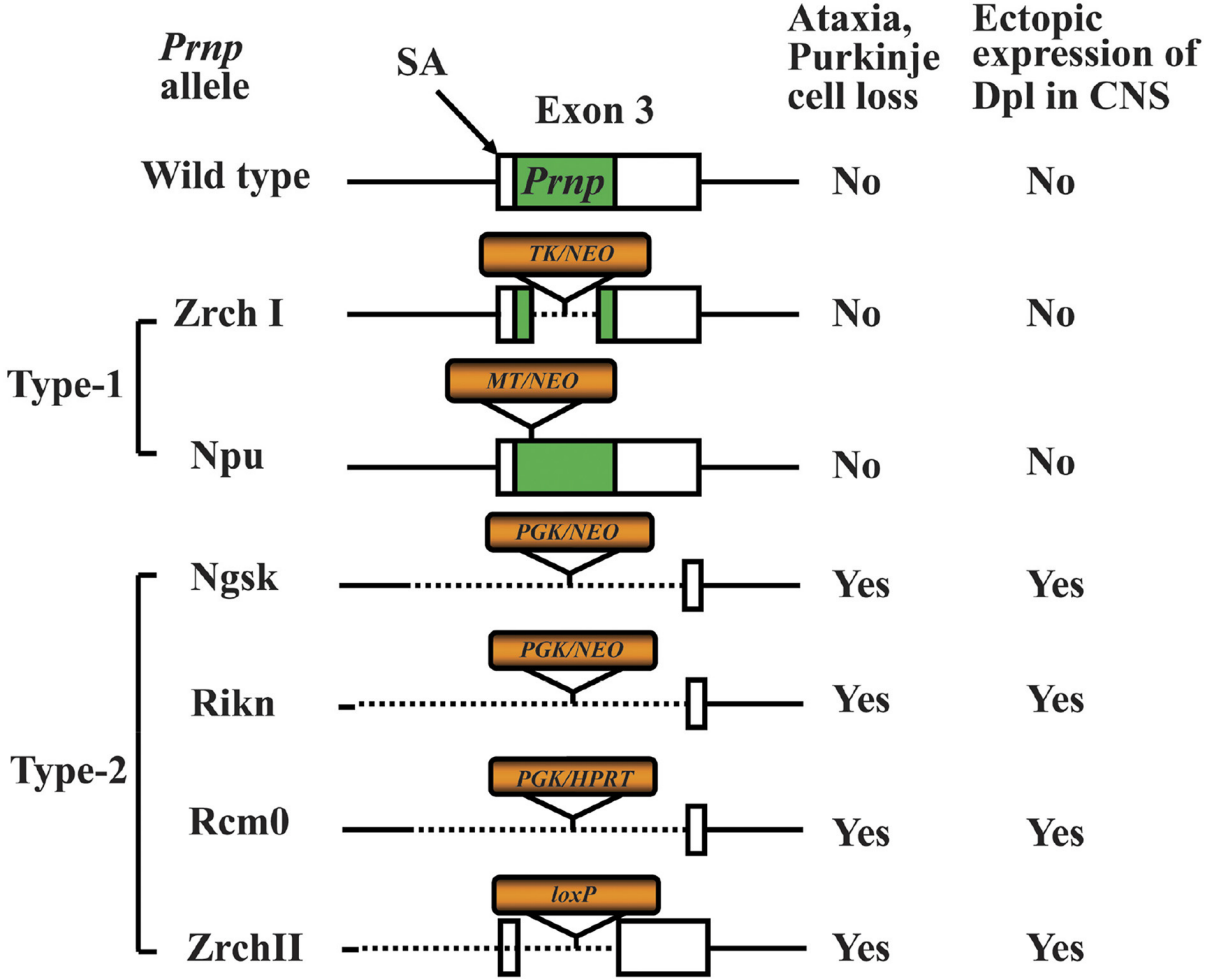
**Knockout constructs of type-1 and type-2 of prion protein gene (*Prnp*)-knockout (*Prnp*^−/−^) mice**. Structures of the constructs used to produce six lines of *Prnp*^−/−^ mice. The *Prnp*^−/−^ mice are divided into type-1 and type-2 *Prnp*^−/−^ mice depending on whether the splicing acceptor of exon 3 is disrupted. The disruption of exon 3 is correlated with the development of late-onset ataxia and Purkinje cell loss, which is induced by ectopic expression of Doppel (Dpl) in the central nervous system (CNS). The structure of wild-type (WT) *Prnp* exon 3 and prion protein (PrP) coding region (green box) is shown at the top. The selection markers are indicated by orange boxes. The presence and absence of the exon 3 splicing acceptor (SA) is correlated with the development of late-onset ataxia. The selection markers were PGK, mouse phosphoglycerate kinase promoter; NEO, neomycin phosphotransferase; HPRT, mouse hypoxanthine phosphoribosyltransferase; TK, human herpes simplex virus type 1 thymidine kinase promoter; MT, mouse metallothionein promoter; loxP, a 34-bp recombination site from phage P1.

One approach to studying protein function is to analyze the effect of knocking out the corresponding gene. In the case of PrP^C^, *Prnp*^−/−^ mice have been widely used for this purpose. Therefore, studies using *Prnp*^−/−^ mice should be mentioned before we discuss *Prnp*^−/−^ cell lines. *Prnp*^−/−^ mice have been used for elucidating the functions of PrP^C^ *via* analysis of the phenotype of *Prnp*^−/−^ mice (Weissmann and Flechsig, 2003). Six lines of *Prnp*^−/−^ mice, designated Zrch I (Bueler et al., 1992), Zrch II (Rossi et al., 2001), Npu (Manson et al., 1994a), Ngsk (Sakaguchi et al., 1996), Rcm0 (Moore et al., 1999) and Rikn (Yokoyama et al., 2001), have been generated (Figure 1). However, there were some discrepancies among the phenotypes of the knockout mice. The first and second knockout lines, Zrch I and Npu, were generated by disrupting the PrP coding region located in exon 3 of the *Prnp* (Bueler et al., 1992; Manson et al., 1994a). Studies on these lines did not show any severe abnormality. Subsequently, Ngsk, Rikn, Rcm0, and Zrch II mouse lines were generated in which the entire coding region and part of intron 2 was deleted (Sakaguchi et al., 1996; Moore et al., 1999; Rossi et al., 2001; Yokoyama et al., 2001). Because of the structure of the targeted *Prnp* allele, intergenic splicing between *Prnp* and the surrounding gene led to ectopic expression of the surrounding gene in the brains of these mice. This prompted the discovery of the gene *Prnd* located 16 kbp downstream of *Prnp*, encoding the prion-related protein Doppel (Dpl) (Moore et al., 1999), which shares ~25% identity with two-thirds of the C-terminal region of PrP. Ectopic expression of Dpl leads to the development of late-onset ataxia in *Prnp*^−/−^ mice. Therefore, the ataxic phenotype of some lines of *Prnp*^−/−^ mice (Ngsk, Rikn, Rcm0, ZrchII) is due to ectopic expression of Dpl (derived from *Prnp*/*Prnd* chimeric mRNAs through intergenic splicing) as a result of the disruption of the splicing acceptor of *Prnp* exon 3 (Moore et al., 1999; Li et al., 2000a; Rossi et al., 2001). In this review article, to discriminate between *Prnp*^−/−^ mice with and without ectopic expression of Dpl, we term the former ataxic *Prnp*^−/−^ mice (Ngsk, Rikn, Rcm0, Zrch II) as type 2, while the latter non-ataxic *Prnp*^−/−^ mice (Zrch I, Npu) as type-1 (Figure 1).

The type-1 *Prnp*^−/−^ mice display no major abnormalities. Indeed, only small abnormalities could be found in type-1 *Prnp*-knockout mice. These include deficits related to neuronal and other cellular functions such as abnormal learning and sleep patterns, olfactory deficits, susceptibility to seizures, abnormal neuromuscular function and depressive disorder as well as abnormal glucose tolerance and deficits related to protection against pathophysiological stress (e.g., abnormal inflammatory responses, decreased protection against viral bacterial infection, accelerated symptoms of experimental autoimmune encephalomyelitis (EAE) and susceptibility to ischemic brain injury (Onodera et al., 2014). In addition, the most consistent phenotype of *Prnp*^−/−^ mice is myelin degeneration (Nishida et al., 1999; Baumann et al., 2007; Bremer et al., 2010). Some of these abnormalities may be due to the genetic background (Steele et al., 2007). Therefore, experiments using backcrossed mice should be performed to confirm whether the observed phenotypes are attributable to PrP. There are also possibilities of synergistic phenotypes in a combination of mouse strain genetic backgrounds and knockout constructs. In addition, the breeding condition of the mice may affect emergence of some phenotypes. For knockout mouse studies, confirmation of findings using mice with distinct knockout constructs and genetic backgrounds in an independent laboratory will be required to firmly establish conclusions regarding PrP function. Indeed, we have recently reviewed the topic of different *Prnp*^−/−^ mouse phenotypes to assess the physiological function of PrP^C^ (Onodera et al., 2014).

## PrP family proteins and their knockout mice

Recently, new members of the PrP family of proteins have been identified; namely, Dpl, encoded by *Prnd*, and Shadoo (Sho), encoded by *Sprn*. These PrP family proteins are all subject to endoproteolytic processing (Mays et al., 2014) and localize to similar membrane environments (Li et al., 2013). Dpl and Sho pose interesting and important questions concerning PrP function. Given that the function of PrP remains elusive, information obtained from comparative structural and functional studies of these PrP family proteins are of great interest. Specifically, complementary, overlapping or interference of function among PrP family members might be found from these analyses.

There are reports indicating functional interaction between PrP and Dpl. It has been shown that the ataxic phenotype observed in type-2 *Prnp*^−/−^ mice can be neutralized by crossing the mice with those overexpressing wild-type mouse PrP (Nishida et al., 1999; Rossi et al., 2001). These findings suggest that PrP has a neuroprotective role and functionally interacts with Dpl. Another report showed that the toxicity of Dpl overexpression in mice and cells is inhibited by PrP expression. In a separate report, using immortalized *Prnp*^−/−^ neuronal cells, a decrease of cellular copper levels brought about by serum deprivation was also inhibited by PrP. However, the inhibitory effect of PrP on decreased copper levels was prevented by Dpl overexpression (Sakudo et al., 2004). A further study demonstrated that endoproteolysis of PrP was affected by Dpl overexpression in *Prnp*^−/−^ cells (Sakudo and Onodera, 2011b). Therefore, PrP-Dpl interaction functions both *in vitro* and *in vivo*.

Dpl is localized in the brain for only a limited duration in the developmental process (Li et al., 2000b), whereas analysis of *Prnd*^−/−^ mice showed that Dpl deficiency did not interfere with embryonic and postnatal development (Behrens et al., 2002; Genoud et al., 2003). Dpl was produced at a late stage of spermatogenesis. Spermatids of *Prnd*^−/−^ mice were reduced in numbers, immobile, malformed and unable to fertilize oocytes, resulting in male sterility. PrP is also expressed in testis, but unlike other cells the PrP is expressed as N-terminally truncated isoforms (Peoc'h et al., 2002) or a C-terminally truncated isoform (Shaked et al., 1999). The C-terminal region of PrP resembles Dpl, suggesting that these proteins share a common function in testis in the case of N-terminally truncated PrP (Peoc'h et al., 2002) or show a complementary function to each other in the case of C-terminally truncated PrP (Shaked et al., 1999). Currently, there is no evidence that Dpl is associated with the pathogenesis of prion diseases. For example, the level of Dpl expression does not correlate with the onset of prion disease (Weissmann and Aguzzi, 1999; Tuzi et al., 2002).

By contrast, there is evidence that Sho is implicated in prion diseases. For example, the expression of Sho decreases with the accumulation of PrP^Sc^ after prion infection (Watts et al., 2007a, 2011). Sho protein levels were decreased in the brains of *Prnp*^a^ and *Prnp*^b^ mice (Westaway et al., 2011), hamsters, meadow, voles and sheep infected with natural and experimental prion strains (Watts et al., 2011). In addition, time course experiments showed that the levels of PrP^Sc^ vs. Sho protein were inversely proportional (Watts et al., 2011). Membrane anchoring and the N-terminal domain of PrP both influenced the inverse relationship between PrP^Sc^ and Sho (Watts et al., 2011). By contrast, increased expression of Sho did not influence prion replication (Watts et al., 2011), suggesting Sho merely acts as a marker for prion disease. Therefore, it remains unclear how Sho contributes to the pathogenesis of prion diseases. Indeed, depletion of Sho appears to be unimportant in terms of triggering prion diseases and in the processing and degradation of PrP^Sc^.

In terms of physiological action, Sho can exhibit neuroprotective properties similar to PrP^C^ (Watts and Westaway, 2007b; Watts et al., 2007a) and share a number of binding partners common with PrP^C^ (Watts et al., 2009). In addition, as Sho has a similar structure to the N-terminal region of PrP (Watts and Westaway, 2007b), the relationships between the structural similarity and functional similarity of PrP and Sho are interesting. *Sprn*^−/−^ mice underwent a subtle alteration of body weight (Passet et al., 2013), which was not noted in the case of double-knockout (*Sprn* and *Prnp*) mice. In addition, *Prnp*/*Sprn* knockout mice survived to over 600 days of age without any severe abnormality, suggesting the existence of a discrete signaling pathway of *Prnp* and *Sprn* to maintain neuronal survival. Sho was also found to be expressed in the trophoblast cells of the placenta (Passet et al., 2012). Comparative transcriptomic analyses performed between E6.5 and E7.5 *Sprn*-knockdown (using RNAi) embryo and their wild-type counterparts suggested that Sho has functions complementary, not necessary overlapping, with those of PrP, associated with cellular movement and hematological system development and differentiation (Passet et al., 2012). Interestingly, the expression profile of *Sprn* in testis and ovary resemble that of *Prnp*. Thus, interaction among three PrP family members may play important roles in reproductive tissues. Although the contribution of PrP family members to embryogenesis is suggested, *Prnp*/*Sprn* knockout mice are healthy and fertile (Daude and Westaway, 2012a; Daude et al., 2012b). Therefore, further studies on reproductive tissues are required to resolve the apparent discrepancy in the data. The topic of Sho is also discussed in detail in a review article in this research topic (Makzhami et al., 2014). As mentioned above, analysis of the phenotypes of knockout mice and comparison of PrP family members does not fully elucidate the functions of PrP. Therefore, other approaches to analyze PrP function are required. Next, we discuss the use of *Prnp*^−/−^ cell lines to study the role of PrP.

## Challenges to investigating PrP^C^ functions *in vitro*

Cells have been classified into approximately 200 different types (Obinata, 2007). Through development and maintenance during adult life, cells are differentiated and obtain characteristics to elicit specialized functions. Differentiation can be chemically induced by various stimuli such as growth factors, cytokines, and hormones (Alberts et al., 2008). Histological location of the cells also influences cell differentiation. In order to study each cell type possessing a specialized function, the cells need to be separated from each other. Examination of large, pure populations of specific cell types is extremely valuable in advancing our understanding of the cells. Primary cultures are composed of a heterogeneous cell population and can be maintained only for a limited period of time. Although PrP^C^ is highly expressed in neurons, primary neuron cultures can be maintained for just a few weeks. Moreover, cells belonging to a rare cell type are often difficult to isolate and culture. For detailed functional gene analysis, an efficient transfection procedure is required. However, the transfection efficiency of primary cultures is generally low, especially when using primary neurons. If gene transfer to a primary culture of neurons or other cell types with low efficiency is necessary, a viral vector is required for the introduction of inducible genes. In this respect, cell lines are useful for detailed analysis of gene function because the transfection efficiency is usually high compared to that of primary cultures. In addition, cell lines can be maintained almost indefinitely.

However, studying pure cell lines has the disadvantage that it can only reflect a cell-autonomous condition, whereas biological processes in an organism involve a heterogeneous cell population. Co-cultures of multiple cell populations may help to mimic the heterogeneous condition. Additionally, because there are a lot of cell types in an organism, it is very difficult to match cell types between cell lines when a comparison is required. Thus, comparisons between a wild-type and a knockout cell line are suboptimal for analyzing a target gene. Alternatively, a cloned knockout cell line should be compared in the absence and presence of the target gene.

## PrP^C^ expression and PrP^C^-interacting proteins

PrP^C^ is expressed in a variety of tissues. Northern blot analysis has shown that the levels of PrP mRNA vary among tissues, with the highest levels found in the brain and placenta; moderate levels in the testis, heart, and lung; and lower levels in the spleen and kidney (Horiuchi et al., 1995; Saeki et al., 1996). In sheep, 3–5 μg PrP^C^/g of tissue is present in the brain, whereas the level of PrP^C^ per gram of tissue is 100 ng in the heart and skeletal muscle, 200 ng in the lung, 40 ng in the spleen, and 3 ng in the liver (Moudjou et al., 2001). Thus, the quantity of PrP^C^ in the brain is 20- to 50-fold higher than in the other tissues. This observation suggests that PrP^C^ plays an important role in several tissues, particularly in the brain.

Recently, it has been shown that PrP^C^ binds a variety of partner molecules. Using radioisotope or enzyme-labeled PrP, interaction of PrP with glial fibrillary acidic protein (GFAP), a NF-E2 related factor 2 (Nrf2), amyloid precursor protein 1 (Aplp1), F-box protein-6, neural F-box protein 42 kDa (NFB42), postsynaptic density 95 kDa (PSD-95)/ SAP-90 associated protein, protein tyrosine phosphatase non-receptor type-21, and predicted protein KIAA0443 was reported (Yehiely et al., 1997). A two hybrid system showed that laminin (Graner et al., 2000), 37-kDa/67-kDa laminin receptor (Gauczynski et al., 2001), 37-kDa laminin receptor precursor (Rieger et al., 1997), heat shock protein 60 kDa (Hsp60) (Edenhofer et al., 1996) and Bcl-2 (Kurschner and Morgan, 1995) all bind PrP. Co-immunoprecipitation experiments were used to identify PrP-interacting molecules, which included prion interactor 1 (Pint1) (Spielhaupter and Schatzl, 2001), synapsin Ib (Spielhaupter and Schatzl, 2001), growth factor receptor-bound protein 2 (Grb2) (Spielhaupter and Schatzl, 2001), and neurotrophin receptor interacting MAGE (melanoma-associated antigens) homolog (NRAGE) (Bragason and Palsdottir, 2005), dystroglycan (Keshet et al., 2000), the neuronal isoform of nitric oxide synthase (nNOS) (Keshet et al., 2000), glucose regulated protein 94 (Grp94) (Capellari et al., 1999), protein disulphide isomerase (Capellari et al., 1999), calnexin (Capellari et al., 1999), calreticulin (Capellari et al., 1999) and zeta-associated protein-70 (ZAP-70) (Mattei et al., 2004). A cross-linking method demonstrated interaction of PrP with tubulin (Nieznanski et al., 2005) and neural adhesion molecule (N-CAM) (Schmitt-Ulms et al., 2001). Complementary hydropathy and pull-down assays were able to show the interaction of PrP with stress inducible protein 1 (STI1) (Martins et al., 1997) and Hsp60 of *Brucella abortus* (Watarai et al., 2003). Intriguingly, PrP interacts with caveolin-1 (Toni et al., 2006), while cross-linking of cell-surface PrP stimulated caveolin-1-dependent interaction with Fyn tyrosine kinase (Mouillet-Richard et al., 2000), resulting in neurite outgrowth and differentiation of neuronal cells (Mouillet-Richard et al., 2000; Pantera et al., 2009). Thus, PrP contributes to the control of the cellular redox state and homeostasis of neuronal cells (Mouillet-Richard et al., 2007). Because Fyn is involved in various signaling pathways, the interaction implies that PrP^C^ has diverse functions. Most interestingly, a wealth of recent studies has established that PrP interacts with Amyloid β protein (Aβ), which is generated by the abnormal processing of the amyloid precursor protein (APP) by β-secretase, β-site APP cleaving enzyme (BACE1) and involved in the pathogenesis of Alzheimer's disease (Larson et al., 2012; Um et al., 2012; Um and Strittmatter, 2013; Dohler et al., 2014). In addition, several reports have shown that PrP^C^ interacts with APP (Yehiely et al., 1997; Kaiser et al., 2012). Several reports have further demonstrated an involvement of PrP in the toxicity of Aβ, although the use of different in *vitro* or transgenic models has yielded contrasting results (Schwarze-Eicker et al., 2005; Laurén et al., 2009; Balducci et al., 2010; Calella et al., 2010; Chung et al., 2010; Kessels et al., 2010; Morales et al., 2010; Ordóñez-Gutiérrez et al., 2013; Gasperini and Legname, 2014). Some groups have also reported that Fyn kinase mediates signal transduction downstream of the PrP^C^-Aβ complex (Larson et al., 2012; Um et al., 2012; Um and Strittmatter, 2013). Because PrP^C^ inhibits BACE1 either by direct interaction (Griffiths et al., 2011) or indirectly without interaction (Parkin et al., 2007; McHugh et al., 2012), reduction of the PrP^C^ level may increase Aβ. Therefore, PrP^C^ may be involved in the pathogenesis of Alzheimer's disease not only by transducing Aβ toxic signals but also *via* regulation of neurotoxic Aβ production. Taken together, most of the interacting proteins are important factors involved in survival, proliferation, differentiation, development, and stress response. However, it should be mentioned that this interaction may depend on the specific cell type and/or the surrounding tissue environment.

**Figure 2:**
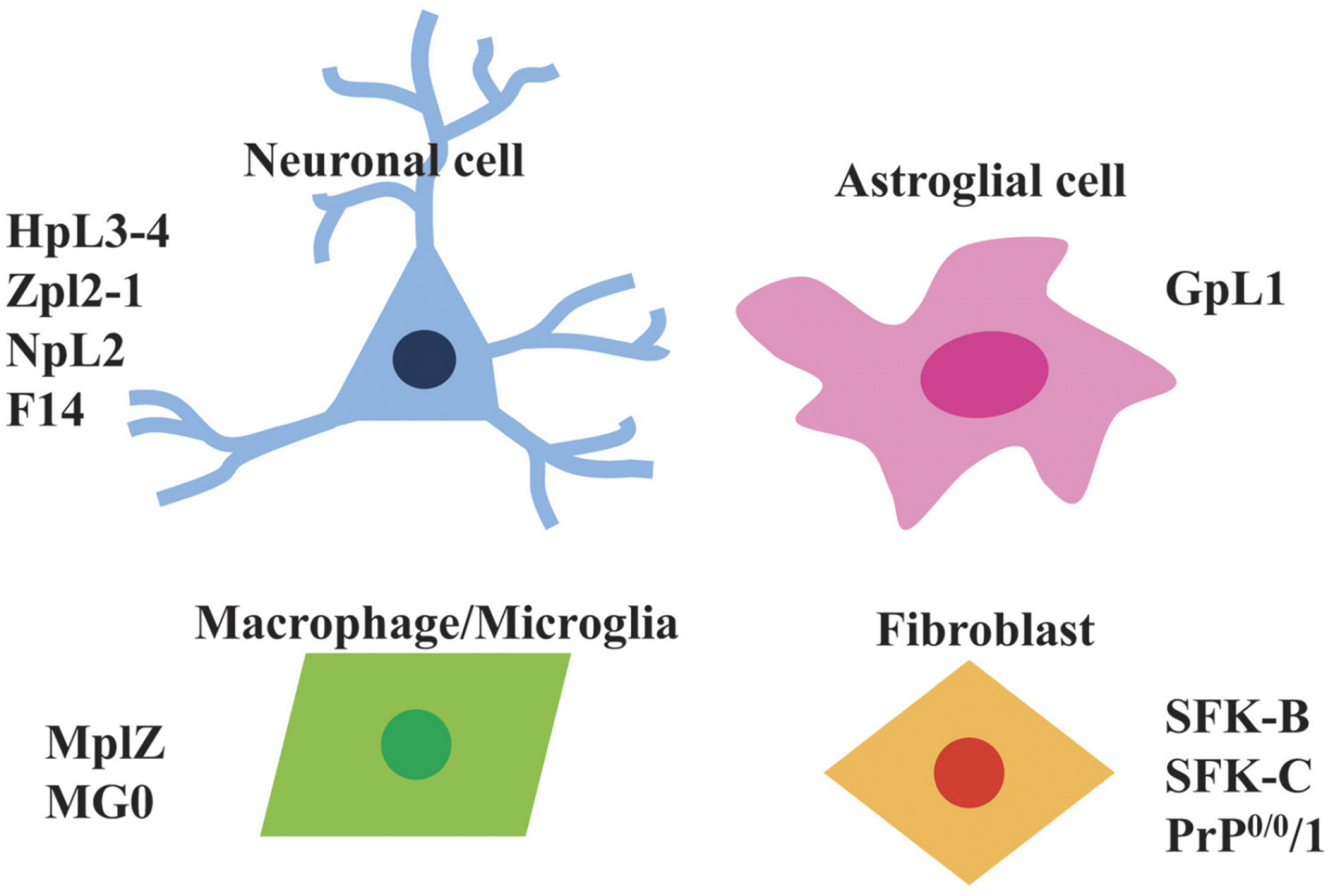
**Types of *Prnp*^−/−^ cell lines that are available**. Currently available *Prnp*^−/−^ cell lines can be divided into four types i.e., neuronal cells (HpL3-4, Zpl2-1, NpL2, F14), astroglial cells (GpL1), macrophage/microglia (MpLZ, MG0), and fibroblasts (SFK-B, SFK-C, PrP^0/0^/1).

Currently, *Prnp*^−/−^ cell lines have predominantly been established from brain as well as fibroblast and macrophage cell lines (Figure 2). Next, we will introduce the *Prnp*^−/−^ cell lines established so far.

## HpL, *Prnp^−/−^* neuronal cell lines

To gain further insights into PrP functions, cell lines (HpL and HW) were established using the gene transfer of oncogenes by our group (Kuwahara et al., 1999). HpL was the first *Prnp*^−/−^ cell line, while HW is the wild-type counterpart. These cell lines were from the hippocampal area of Rikn *Prnp*^−/−^ mice and *Prnp*^+/+^ mice using a target for infection with a recombinant retrovirus vector containing the simian virus 40 (SV40) T-antigen gene (Ryder et al., 1990). The virus vector was used for transfection of ψ2 packaging cells, which are stable cells containing all of the structural retroviral protein genes (*gag, pol*, and *env*). The retrovirus particles produced from the transfected ψ2 packaging cells were used for immortalization by introduction of the SV40 large T antigen gene. The hippocampal primary cultures derived from *Prnp*^−/−^ and *Prnp*^+/+^ mice were infected with the above retrovirus, then incubated, selected, expanded and finally subjected to limiting dilution to obtain cloned cell lines. Three *Prnp*^−/−^ cell lines, HpL2-1, HpL-3-2 and HpL3-4, and 3 *Prnp*^+/+^ cell lines HW8, HW9, HW19, were thereby obtained (Kuwahara et al., 1999).

The HpL and HW cells were exposed to dibutyl cyclic AMP (dcAMP) at 0.4–6 mM, retinoic acid (RA) at 0.1–10 μM, and phorbol 12-myristate 13-acetate (PMA) at 160–1600 nM, for up to 10 days of culture. These compounds all induced morphological changes, i.e., elongation of neurite-like processes, in both HpL and HW cells. Among the tested compounds, the morphological change was best observed by stimulating the HpL and HW cells in medium with dcAMP or PMA without fetal calf serum (FCS). To further characterize the cell types of the cell lines, reverse transcription polymerase chain reaction (RT-PCR) was applied to monitor the expression of neurofilament genes. Treatment with PMA resulted in the amplification of the predicted size products corresponding to neurofilament (NF)-68K and NF-200K genes. The NF-68K gene was transcribed in all 3 HpL and 3 HW cell lines with or without PMA treatment. After treatment with PMA, the NF-200K gene was transcribed in all three HW cell lines and in only one of the HpL cell lines (HpL2-1). The transcription of GFAP, a glial cell marker, was not detected in HpL or HW cells. Taken together, the results suggest that all six of these cell lines belong to the neuronal precursor cell lineage.

Next, the characteristics of HpL cells were compared to those of HW cells. The most remarkable feature of HpL cells by comparison to HW cells was observed when the serum was removed from the culture medium (Kuwahara et al., 1999). Serum deprivation caused comparable morphological changes within 1 h in all cell lines whether or not they were treated with dcAMP. However, within 4 days the HpL cells had died in the serum-free culture. The process started by the rounding up of the cells, cytoplasmic condensation and neurite retraction. By contrast, all three HW cell lines maintained their structural integrity under the same culture conditions. DNA fragmentation, a characteristic of apoptosis, was firstly observed at 12 h and peaked at 24 h after serum deprivation in the HpL cells, whether or not they were treated with PMA or cAMP. However, no such DNA fragmentation was detected in any of the HW cells after serum deprivation. Cultured cells in serum-free medium for 6, 12, or 24 h were ethanol-fixed and stained with propidium iodide before analysis by flow cytometry (Sakudo et al., 2003a). A significant peak in the fluorescence low-intensity area (hypodiploid cells), which corresponds to the fraction of apoptotic cells, was detected only in the samples from HpL cells at 6, 12, and 24 h after serum withdrawal. Because HpL3-4 cells started to generate superoxide anion at 6 h after serum deprivation, superoxide anion generation seems to induce apoptosis of the HpL3-4 cells (Sakudo et al., 2003b). To investigate whether the abnormality observed in HpL cells was due to the absence of PrP, the HpL cell lines (HpL2-1 and HpL3-4) were transfected with PrP expression vector. Cells transfected with PrP-expressing vector, designated HpL2-1TR and HpL3-4TR, survived under serum-free conditions (Kuwahara et al., 1999). In contrast, HpL2-1 and HpL3-4 cells transfected with empty vector showed apoptotic features similar to those of HpL2-1 and HpL3-4 cells under serum-free conditions. These results suggest that HpL cells are susceptible to serum deprivation and that apoptotic cell death is suppressed by reintroduction of *Prnp*. Moreover, levels of superoxide anion generated from HpL3-4 cells after serum deprivation were suppressed by reintroduction of *Prnp* (Sakudo et al., 2003b). Therefore, these results suggest that PrP functions by displaying anti-oxidative and anti-apoptotic activity. Recent studies have shown that the anti-apoptotic activity of PrP is species specific, as indicated by evidence that apoptosis was suppressed by mouse PrP but not by bovine or hamster PrP (Wu et al., 2008). Moreover, as serum-free supernatants of HW cell cultures could not rescue the HpL cells from apoptosis, molecules secreted from HW cells do not appear to be involved in the neuroprotective mechanism of PrP (Kuwahara et al., 1999). In addition to the cell death, HpL cell lines show significantly shorter neurite extension in comparison with HW cell lines after treatment with PMA, suggesting that PrP is involved not only in survival but also in differentiation (Kuwahara et al., 1999).

Recently, in addition to our group, several other groups have independently confirmed the neuroprotective function of PrP using the above-mentioned PrP-deficient cell lines or other cell lines. Kim et al. reproduced our findings using HpL3-4 cells, and detected significantly greater changes in the calcium ion level, transmembrane potential and cytochrome c level in the mitochondria of HpL3-4 cells over those observed in *Prnp*-reintroduced HpL3-4 cells after serum deprivation (Kim et al., 2004). Mange et al. also confirmed that cell viability was increased by the reintroduction of *Prnp* into HpL3-4 cells (Mange et al., 2004). By contrast, Christensen et al. confirmed a moderate but significant cytoprotective effect of PrP in serum-deprived HpL3-4 cells, but concluded the degree of neuroprotection offered by PrP was either not physiologically relevant or that the cell culture systems cannot adequately model the situation *in vivo* (Christensen and Harris, 2008). However, we believe the HpL3-4 cells used in these experiments were not appropriate for this analysis because they did not express neuronal markers (i.e., neuronal nuclear antigen (NeuN), microtubule-associated protein 2 (MAP2) or NF-L) (Christensen and Harris, 2008). This opinion is also supported by the evidence that *Prnp*-*Prnd* chimeric mRNA transcribed from the *Prnp* promoter was not detected by RT-PCR and immunofluorescence analysis in the cells used by Christensen et al. By contrast, our results were confirmed by *Prnp-Prnd* chimeric mRNA expression in HpL3-4 cells (Sakudo et al., 2005b).

HpL3-4 cells are susceptible to various forms of stress. For example, Vassallo et al. showed HpL3-4 cells are prone to cell death induced by 3-morpholinosydnonimine (SIN-1) (Vassallo et al., 2005). Vassallo et al. further investigated the signal cascade of apoptosis induced by serum deprivation and SIN-1, and showed that recruitment of phosphatidylinositol 3-kinase by PrP contributes to cellular survival under conditions of oxidative stress imposed by SIN-1 or serum deprivation in HpL3-4 cells. *Prnp*-transfected HpL3-4 cells show autophagy after amyloid β treatment. Amyloid β_1−42_ (Aβ_42_) enhanced autophagic flux, such as the formation of autophagic vacuoles, was observed in HpL3-4 cells in which *Prnp* was reintroduced. However, autophagy was impaired in HpL3-4 cells transfected with empty vector (Nah et al., 2013). In addition, PrP-dependent recruitment of Beclin1 (BECN1) into lipid rafts was observed in the HpL3-4 cell line. Because BECN1 is associated with phosphatidylinositol 3-kinase catalytic subunit type 3 (PIK3C3), there will be recruitment of BECN1-PIK3C3 complex into the lipid raft, which is an essential step for autophagy. The observed autophagy could be due to interaction of PrP and BECN1. Therefore, studies using HpL3-4 cells revealed that PrP is involved in Aβ_42_ induced autophagy.

HpL3-4 cells were also susceptible to a monomeric, highly α-helical form of PrP known as toxic PrP (Zhou et al., 2012). Toxic PrP was identified as a recombinant form of PrP that was subjected to denaturation, fractionation and dilution refolding. However, there is no information on the comparative effect of toxic PrP on PrP-expressing and non-expressing HpL3-4 cells. Similarly, in another report, although treatment with Aβ_42_ showed a cytotoxic effect and increased levels of reactive oxygen species (ROS) in HpL3-4 cells, there was no comparison with the HpL3-4 counterpart in which *Prnp* was reintroduced (Hyeon et al., 2012).

It has also been reported that treatment of HpL3-4 cells with hydrogen peroxide increases intracellular calcium levels in a PrP expression dependent manner, suggesting PrP functions as a sensor of hydrogen peroxide (Krebs et al., 2007). Therefore, it appears that PrP^C^ plays an important role in anti-oxidative stress. As such, the signaling cascade and oxidative metabolism were investigated in HpL3-4 cells after serum deprivation (Sakudo et al., 2003a,b). The studies revealed decreased expression of anti-apoptotic proteins Bcl-2 and Bcl-x_L_ in HpL3-4 cells during apoptosis induced by serum deprivation. Suppression of apoptosis by overexpression of Bcl-2 and Bcl-x_L_, suggested that cell death in this model system is apoptotic, and Bcl-2 and Bcl-x_L_ play important roles in the apoptosis of HpL3-4 cells (Sakudo et al., 2003a). Furthermore, HpL3-4 cells subjected to serum deprivation showed a more marked decrease in intracellular copper concentration, greater increases of intracellular superoxide anion and caspase-3/9 activation, and a smaller increase in intracellular hydrogen peroxide levels by comparison to HpL3-4 cells in which *Prnp* was reintroduced (Sakudo et al., 2003b). HpL3-4 cells also showed a decrease of cellular superoxide dismutase (SOD) activity and cellular copper content compared to HpL3-4 cells expressing PrP (Sakudo et al., 2003b). By contrast, levels of *Prnp*-*Prnd* chimeric mRNA and *Prnd* mRNA were unchanged in HpL3-4 cells after serum deprivation (Sakudo et al., 2005c). Higher coxsackievirus B3 and poliovirus production in HpL3-4 cells showed that PrP may be involved not only in the inhibition of virus replication but also anti-apoptotic functions against virus-induced apoptosis (Nakamura et al., 2003b; Baj et al., 2005). Taken together, our studies of HpL cell lines have revealed that PrP plays important roles in differentiation, proliferation and cell survival (i.e., roles in anti-apoptosis, anti-oxidative and anti-viral effect *via* regulating a variety of cell signal cascades). The results obtained from the study of HpL cell lines are consistent with those derived from other studies using primary cultures and other cell lines, which also showed the involvement of PrP in neuroprotection and differentiation (Milhavet and Lehmann, 2002; Roucou et al., 2004).

Studies using HpL cells have contributed to identifying PrP-interacting molecules or PrP-regulated genes because HpL does not express PrP. Moreover, *Prnp*-transfected HpL cells are an ideal counterpart for PrP analysis. Zafar et al. used HpL3-4 cells in combination with STrEP-Tactin chromatography and quadrupole time-of-flight tandem mass spectrometry (Q-TOF MS/MS) analysis to search for PrP-interacting proteins and identified 28 new hits (Zafar et al., 2011). Among them, Rab7a, a regulator of vesicular transport located in a specific intracellular compartment (early to late endosome) and involved in vesicle biogenesis and vesicle fusion to lysosomes, was found to interact with PrP and regulate PrP trafficking in HpL3-4 cells. Furthermore, 23 PrP-interacting proteins were identified by using deletion mutants of PrP [PrP(Δ23-230)], which lack the N-terminal signal peptide sequence and C-terminal glycosylphoshatidylinositol (GPI) anchor site (Zafar et al., 2014). Among them, one novel protein (pyruvate kinase isozymes M1/M2) (PKM2) was found to bind PrP(Δ23-230). In addition, PrP(Δ23-230) exhibits reduced anti-apoptotic activity against staurosporine-induced cell stress with higher expression of PKM2 than full length PrP in HpL3-4 cells, suggesting that PrP(Δ23-230) regulates PKM2 and renders the cells susceptible to stress.

Taking advantage of the absence of *Prnp*, HpL3-4 cells have been used for the analysis of exogenous PrP. Hachiya et al. reported an N-terminal PrP fragment and C-terminal fragment showed distinct intracellular distribution using transfection of double-labeled fluorescent PrP into HpL3-4 cells (Hachiya et al., 2004). The N-terminal PrP fragment was associated with microtubules and had an intracellular localization, but did not co-localize with any intracellular organelle markers.

## Other *Prnp^−/−^* neuronal cell lines

As described earlier, PrP exhibits a neuroprotective effect against apoptosis induced by serum deprivation in HpL3-4 cells, which are derived from type-2 *Prnp*^−/−^ mice (Rikn) with ectopic Dpl expression. To investigate whether PrP inhibits apoptotic neuronal cell death in the absence of Dpl, neuronal cell lines were established from the brain of type-1 *Prnp*^−/−^ mice (Zrch I), which did not show ectopic Dpl expression (Nishimura et al., 2007). The results showed that reintroduction of *Prnp* potently inhibited serum-withdrawal apoptotic cell death in a Zrch I neuronal *Prnp*^−/−^ cell line (NpL2). Moreover, PrP expression was found to upregulate cellular SOD activity in NpL2 cells. Therefore, we concluded that ectopic Dpl production did not affect the anti-apoptotic and anti-oxidative functions of PrP. Thus, it was confirmed that the presence of PrP is directly correlated with protection against oxidative stress.

Kim et al. established neuronal cell lines from hippocampal neurons of Zrch I *Prnp*^−/−^ mice by lipofection of SV40 large T antigen-expressing vector and found higher proliferation of neuronal cell lines from *Prnp*^−/−^ mice (Zpl) than those derived from *Prnp*^+/+^ mice (ZW) (Kim et al., 2005). Zpl cells do not express Dpl because the cells are derived from type 1 *Prnp*^−/−^ mice (Zrch I). Moreover, Zpl cells showed higher levels of apoptosis and autophagy compared to ZW cells after serum deprivation (Oh et al., 2008). The neuroprotective activity of PrP in Zpl cells was confirmed by reintroduction of *Prnp* into Zpl cells. Cleaved caspase-3 levels for apoptotic index and LC3-II levels for autophagy index were correlated with the expression of PrP. Furthermore, PrP(Δ53-94), octapeptide repeat (OR)-deficient PrP, did not display a protective activity in Zpl cells, indicating the importance of the OR region in the neuroprotective activity of PrP. These results are consistent with the HpL3-4 studies using deletion PrP mutants as described in the following section.

The F14 neuronal cell line is another *Prnp*^−/−^ derived cell line, obtained by fusion of *Prnp*^−/−^ cerebellar cells and mouse neuroblastoma cells (Holme et al., 2003). The cells were used for investigating the effect of PrP mutations on PrP membrane orientation, although the characteristics of PrP-expressing cells and PrP-deficient cells have not been compared.

Although not *Prnp*-knockout cells, Loubet et al. used the 1C11 cell line, a neuroepithelial progenitor for shRNA (short hairpin RNA)-mediated knockdown of *Prnp*, to investigate the gene silencing effect of *Prnp* (Loubet et al., 2012). 1C11 cells lack neuron-associated functions and acquire, upon differentiation, the functions of serotogenic or noradrenergic neuronal cells associated with cytoskeleton remodeling along neuritogenesis. The results showed that *Prnp* knockdown caused impaired neuronal polarization with inhibition of the initial sprouting of neurite. Further analysis of the signaling pathway suggests that PrP^C^ contributes to neurite polarization by modulating integrin interactions with the extracellular matrix.

## Astroglial *Prnp^−/−^* cell lines

PrP^C^ is expressed not only in neuronal cells but also in non-neuronal cells such as glial cells. Therefore, studies on PrP^C^ functions using non-neuronal cell lines are necessary. To address this issue, the *Prnp*^−/−^ astroglial cell line GpL1 from hippocampal cells of Zrch I *Prnp*^−/−^ mice were established by retrovirus-mediated SV40 large T antigen-immortalization using a retrovirus vector employing methods described above (Nishimura et al., 2008). GpL1 cells were confirmed to express GFAP but not MAP-2, indicating that GpL1 is an astroglial cell line. Transfection of *Prnp* suppressed cell death in GpL1 cells under serum-free conditions. This finding is similar to the results of *Prnp*^−/−^ neuronal cell lines HpL3-4 and NpL2. The *Prnp*-transfected GpL1 cells (GpL1-PrP) showed increased SOD activity compared to control GpL1 cells transfected with empty vector (GpL1-EM). Thus, this study extended the notion that PrP^C^ prevents apoptosis by its anti-oxidative function in not only a *Prnp*^−/−^ neuronal cell line but also a *Prnp*^−/−^ astroglial cell line after serum withdrawal.

In addition to PrP^C^ function in independent cells, we have investigated whether PrP expression on the cell surface of astroglia influences neuronal survival because neurons and astroglia are closely coupled in their metabolic activities (Verkhratsky et al., 2014). Thus, the effect of PrP expression on cell viability of NpL2 cells under co-culture with GpL1-EM or GpL1-PrP cells in the absence of serum was investigated (Onodera and Sakudo, unpublished results). For co-culture experiments, GpL1-EM and GpL1-PrP cells were prepared and replaced with a serum-free DMEM. NpL2-EM and NpL2-PrP cells were plated onto coverslips, which were then transferred to a dish containing GpL1-EM or GpL1-PrP cells. After 4 days of co-culture, the coverslips were retracted and cell viability measured. The results showed that the NpL2 cell viability was significantly increased under co-culture with GpL1 cells transfected with *Prnp* (GpL1-PrP) compared to GpL1 cells transfected with empty vector (GpL1-EM). Similar results were observed when we used both NpL2 cells transfected with *Prnp* (NpL2-PrP) and NpL2 cells transfected with empty vector (NpL2-EM). These findings suggest that the neuronal cell viability depended on the PrP expression in feeder astroglia in the co-culture system. Taken together, astroglial cell lines are useful for revealing the mechanism of PrP^C^ not only in independent astroglial cells but also in the function of the neuron-glia relationship.

## Macrophage/microglia *Prnp^−/−^* cell lines

Recent studies have shown that cells of the immune system, such as macrophages, dendritic cells (DCs) or lymphocytes, can act as a replication site or as a reservoir for prions (Aucouturier and Carnaud, 2002). Follicular dendritic cells (FDCs) in the germinal centers of lymphoid organs are reported to be sites of PrP^Sc^ accumulation (Mcbride et al., 1992; Hill et al., 1999; Aucouturier and Carnaud, 2002). Current data suggest that the prion agent might be acquired by migratory DCs and macrophages (Maignien et al., 2005). However, the function of PrP^C^ in macrophages remains unclear.

Macrophages express PrP^C^ at a very low level. To study PrP^C^ functions in macrophage, the mouse bone marrow-derived macrophages (BMM) from FVB/N *Prnp*^+/+^ and Zrch I *Prnp*^−/−^ mice were transformed with a replication-defective retrovirus encoding SV40 large T antigen by a similar method previously used for the establishment of HpL, NpL, and GpL cell lines (Uraki et al., 2010). The cells were then selected and cloned. Among the obtained clones, we used MWF3-3 (from FVB/N *Prnp*^+/+^ mice) and MplZ4-3 (from Zrch I *Prnp*^−/−^ mice) for comparison, because these cells expressed macrophage specific proteins (F4/80 and MOMA-2) and displayed phagocytotic properties. Because it was previously reported that *Prnp*^−/−^ neuronal HpL and NpL cells and astroglial GpL cells are sensitive to oxidative stress in serum-free conditions, MWF3-3 and MplZ4-3 cells were subjected to serum deprivation. The MplZ4-3 cells died after withdrawal of serum, whereas most MWF3-3 cells survived under the same culture conditions. These findings suggest that PrP^C^ increases the survival rate of macrophages.

With regard to the characteristics of the *Prnp*^−/−^ macrophage cell line, we have analyzed their cell morphology and phagocytotic activity. The *Prnp*^−/−^ macrophage cell line (MplZ4-3) showed shorter pseudopodium extension and less phagocytotic activity of latex beads than a *Prnp*^+/+^ macrophage cell line (MWF3-3). These findings are consistent with the results using peritoneal macrophages from *Prnp*^−/−^ and *Prnp*^+/+^ mice using latex beads (Nitta et al., 2009). However, the findings are inconsistent with a previous study showing that primary cultures of Zrch I *Prnp*^−/−^ macrophages have increased rates of phagocytosis of zymosan particles, suggesting PrP negatively regulates this process (De Almeida et al., 2005). One possible explanation for this discrepancy may be the different mouse strains used in these experiments. Whereas the previous report examined primary cells derived from C57BL/6, we used a macrophage cell line derived from FVB/N. Similarly, *Prnp*^−/−^ mice are resistant to infection by *Brucella abortus*, which is supported in the report by De Almeida et al. implying that the deletion of PrP contributes to host defense against bacterial infection (Watarai et al., 2003). However, this was not confirmed in the case of *Brucella suis* in another laboratory (Fontes et al., 2005). In addition, a recent report by Nuvolone et al. suggests that the increased phagocytosis of apoptotic cells, which was reported as attributable to the absence of *Prnp* in *Prnp*^−/−^ mice, has been shown instead to be caused by differences in a linked locus encoding signal regulatory protein-α (*Sirpa*) (Nuvolone et al., 2013). Thus, further studies would be required for investigating whether *Sirpa*-polymorphism influences the responses to zymosan or phagocytosis of latex beads.

Microglia are the primary immune cells of the central nervous system (CNS), and are highly similar to peripheral macrophages. These cells play important roles as the major inflammatory cell type in the brain, and respond to pathogens and injury and contribute to destroying pathogens as well as removing damaged cells. Several studies have indicated that microglia are implicated in the pathogenic events of prion diseases (Williams et al., 1994; Brown, 2001b). However, the pathophysiological function of PrP^C^ in microglia remains unclear. Iwamaru and Kitani et al. obtained the MG0 *Prnp*^−/−^ microglial cell line, which is derived from the brains of Rikn *Prnp*^−/−^ mice (Iwamaru et al., 2007). To immortalize these microglial cells for establishing the MG0 cell line, *c*-*myc*-containing retroviral vector was used. The MG0 cells were confirmed to be positive for the microglial markers Mac-1 and F4/80 and negative for the astroglial marker GFAP and neuronal marker MAP2. Moreover, these cells were shown to have phagocytotic activity and to produce inflammatory cytokines, such as tumor necrosis factor α, interleukin-1α and interleukin-6, when stimulated with lipopolysaccharide. However, there are no reports on the comparison between MG0 and the wild-type counterpart MG6 cell line or PrP-overexpressed microglial cell line MG20. Persistent infection of scrapie and BSE in these cells has been reported. Further analysis of PrP function in microglia using the MG0 cell line is keenly awaited.

## Fibroblast *Prnp^−/−^* cell lines

Satoh et al. established skin fibroblast *Prnp*^−/−^ cell lines (SFK) derived from continuous cultures of abdominal skin explants of Ngsk *Prnp*^−/−^ mice. SFK cells showed decreased expression of Ras- and Rac-related proteins compared to *Prnp*^+/+^ skin fibroblast cell lines (SFH) (Satoh et al., 1998, 2000), while heat shock proteins were expressed at the same level in SFK and SFH cells in the presence and absence of heat stress (Satoh et al., 1998). Other groups established *Prnp*^−/−^ fibroblasts (PrP^0/0^/1 cell line) from Ngsk *Prnp*^−/−^ mice by immortalization with the chemical mutagen 3-methylcholanthrene (Prcina et al., 2010). The proliferation rate of PrP^0/0^/1 cells expressing human PrP was lower those of non-transfected PrP^0/0^/1 cells, and was negatively correlated with the PrP expression level. However, the authors also stated that these observations may be due to the antibiotic selection marker on the vector.

## Mechanisms by which PrP prevents apoptosis in the HpL cell line

**Figure 3:**
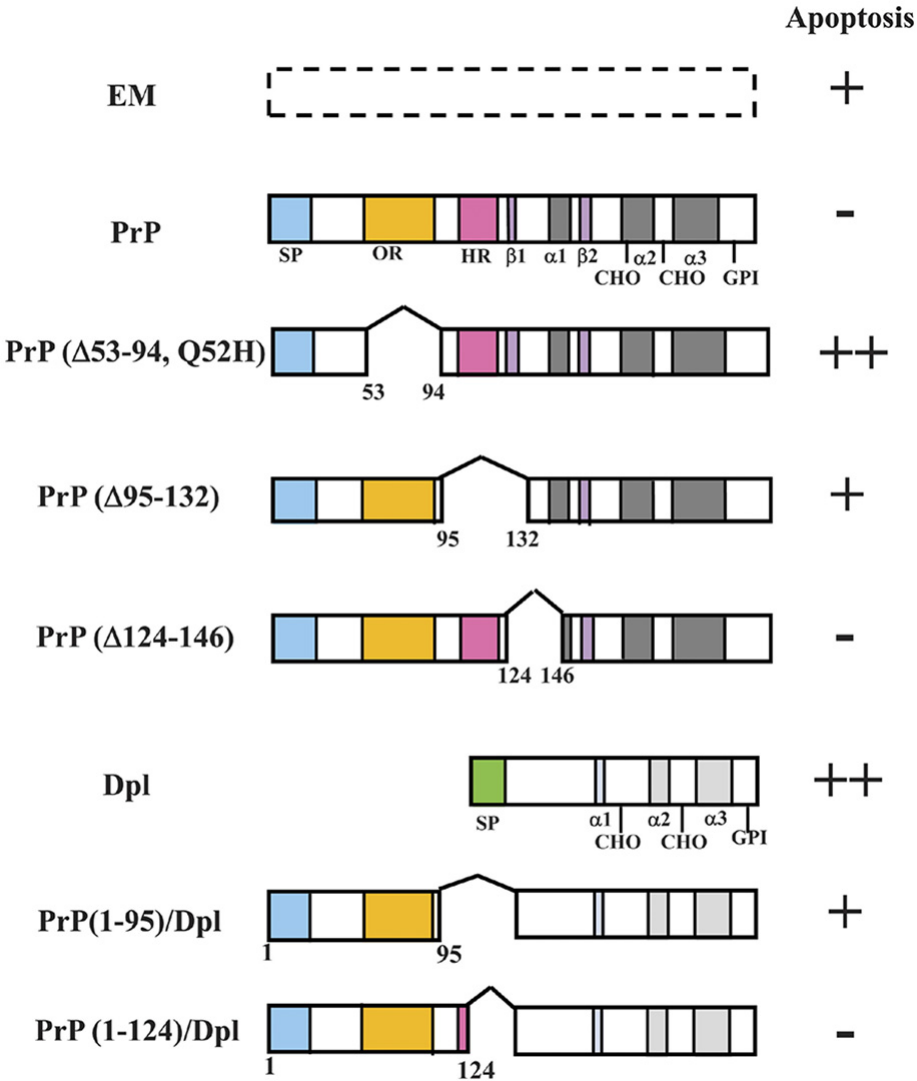
**Schematic representations of PrP deletion mutants or PrP-Dpl fusion proteins and *Prnp*^−/−^ cells (HpL3-4) expressing these mutants that undergo apoptosis under serum deprivation**. Schematic representations of deletion mutants of mouse PrP and PrP-Dpl fusion protein are shown. Mutants of mouse PrP [PrP: wild-type PrP; Δ#1, PrP(Δ53-94, Q52H); Δ#2, PrP(Δ95-132); Δ#3, PrP(Δ124-146)] were prepared using restriction digestion/ligation. Schematic locations of the deletions as compared with the wild-type protein are shown by a space within the bar next to the indicated protein. Dpl lacks sequences homologous to the octapeptide repeat (OR) and hydrophobic regions (HR) of PrP. ORDpl [PrP(1-95)-Dpl] is Dpl fused with amino acid residues 1-95 of PrP containing the OR. ORHRDpl [PrP(1-124)-Dpl] is Dpl fused with amino acid residues 1-124 of PrP containing the OR and N-terminal half of HR. The numbers refer to the amino acid residues in the mouse PrP sequence or Dpl sequence. The Asn-linked glycosylation sites (CHO), signal peptide sequence (SP), octapeptide repeat region (OR) and hydrophobic region (HR) are shown. The regions forming an α-helix secondary structure are shown as α1-α3, whereas those forming a β-sheet are shown as β 1 and β 2. Both PrP and Dpl have a glycosylphosphatidylinositol (GPI) anchor attached to its C-terminus. HpL3-4 cells expressing wild-type PrP (PrP), PrP(Δ53-94, Q52H) (Δ#1), PrP(Δ95-132) (Δ#2), PrP(Δ124-146) (Δ#3), Dpl (Dpl), PrP(1-95)-Dpl (ORDpl), PrP(1-124)-Dpl (ORHRDpl) or the empty vector alone (EM) were serum-deprived. The rate of apoptosis of the cells after serum deprivation for 24 h is shown on the right. The number of pluses (+) indicates the degree of apoptosis. Minus (−) indicates a lesser degree of apoptosis. Updated from Figure 4 in Sakudo et al. (2006) and Figure 3 in Sakudo et al. (2007b) with permission from Bentham Science Publishers, Ltd. and John Wiley & Sons, Inc., respectively.

One way to identify the important regions of PrP is to generate partially deleted or mutated forms of PrP and transfect them into the *Prnp*^−/−^ cell line. Endogenous PrP expression in the *Prnp*^+/+^ cell line may interfere with these experiments. Hence, the *Prnp*^−/−^ cell line is particularly useful for this approach. Here, we introduce our studies using HpL3-4 cells for investigating the important regions for PrP function (Figure 3).

PrP contains disulfide links (S–S), Asn-linked glycosylation sites (CHO), a signal peptide sequence (SP) and a GPI anchor attached to its C-terminus (Brown, 2001a). In addition, PrP^C^ contains two domains evolutionarily conserved among mammals; viz., the OR (amino acid residue 51–90 in mouse PrP) and the hydrophobic region (HR) (amino acid residue 112–145 in mouse PrP) domains.

OR, which is composed of five octapeptide repeats, P(Q/H)GGG(G/-)WGQ, is highly conserved among mammals and can selectively bind copper (Hornshaw et al., 1995; Brown et al., 1997; Miura et al., 1999; Viles et al., 1999; Kramer et al., 2001). Zinc, manganese, and nickel also bind to PrP but with lower affinity (Pan et al., 1992; Brown et al., 2000; Jackson et al., 2001). The binding of metals occurs *via* histidine residues located in the OR domain. The binding of copper to PrP is thought to be indispensable for the SOD activity of PrP (Brown et al., 1999; Cui et al., 2003). In addition, as metals such as copper are essential for myelin formation and maintenance (Skripuletz et al., 2008; Benetti et al., 2010), regulation of copper by PrP^C^ may be involved in demyelination of *Prnp*^−/−^ mice (Nishida et al., 1999; Baumann et al., 2007; Bremer et al., 2010).

HR, which contains the highly conserved sequence motif AGAAAAGA (Gasset et al., 1992; Schatzl et al., 1995), has been shown to be critical for PrP topology in the endoplasmic reticulum membrane (Hegde et al., 1998), STI1 binding (Zanata et al., 2002) as well as normal metabolic cleavage (Chen et al., 1995; Mange et al., 2004) such as α-cleavage and β-cleavage.

To investigate the roles of OR and HR in the anti-apoptotic function of PrP, several deletions within these domains of mouse PrP or fusions with mouse PrP and Dpl have been made (Figure 3). The anti-apoptotic function of three PrP deletion constructs and PrP-Dpl fusion constructs as well as the control constructs was tested following stable transfection into *Prnp*^−/−^ neuronal cells HpL3-4 [HpL3-4 cells expressing wild-type PrP (PrP: HpL3-4-PrP), PrP(Δ53-94, Q52H) (Δ#1: HpL3-4-Δ#1), PrP(Δ95-132) (Δ#2: HpL3-4-Δ#2), PrP(Δ124-146) (Δ#3: HpL3-4-Δ#3), PrP(1-95)-Dpl (ORDpl: HpL3-4-ORDpl), PrP(1-124)-Dpl (ORHRDpl: HpL3-4-ORHRDpl) or empty vector alone (EM: HpL3-4-EM)]. Western blotting confirmed that the cell clones expressed the deletion mutants and fusions at similar levels to those of clones expressing the full-length protein. In the absence of serum, HpL3-4 cells undergo cell death with features of apoptosis, whereas *Prnp*^−/−^ cells transfected with *Prnp* are resistant to serum deprivation (Kuwahara et al., 1999; Sakudo et al., 2003b, 2005a). Serum deprivation showed that the control transfectants (HpL3-4-EM) underwent rapid cell death, while the full-length PrP-transfected cells were significantly protected. In contrast, the cells expressing OR-deficient PrP (HpL3-4-Δ#1) showed higher levels of cell death compared with HpL3-4-EM cells. Interestingly, the cells expressing the N-terminal half of HR-deficient PrP (HpL3-4-Δ#2) underwent cell death at equivalent levels to HpL3-4-EM cells. In contrast, the cells expressing the C-terminal half of HR-deficient PrP (HpL3-4-Δ#3) underwent cell death at equivalent levels to HpL3-4-PrP cells. The cells expressing Dpl fused to OR (HpL3-4-ORDpl) died at slightly higher levels compared to HpL3-4-EM cells, while the cells expressing Dpl fused to OR and N-terminal half of HR (HpL3-4-ORHRDpl) displayed elevated levels of survival over HpL3-4-EM cells (Lee et al., 2006).

Therefore, the data suggest that the anti-apoptotic function of PrP^C^ can be regulated by not only the OR domain but also the N-terminal half of HR. In addition, a fusion protein containing OR of PrP did not protect the cells against stress, whereas extending the fusion to the N-terminal half of HR did provide some protection. These findings support the notion that both the OR and HR are important domains for PrP function. Moreover, measurement of cellular SOD activity showed that OR and the N-terminal half of HR are necessary for the activation of cellular SOD in HpL3-4 cells (Sakudo et al., 2005c). Therefore, regulation of the anti-oxidative defense systems, such as SOD activity, by PrP suggests this domain contributes to the anti-oxidative and anti-apoptotic activity during serum deprivation in HpL3-4 cells.

Mouse PrP is cleaved at the β site located in OR and near His96 as well as the α site located at position 111/112 (Mange et al., 2004). A PrP fragment was immunocaptured from HpL3-4 cells expressing wild-type PrP using an antibody recognizing the N-terminal region of PrP. This fragment mainly includes the N-terminal portion of the protein generated after cleavage at the β site. Interestingly, β cleavage was observed after oxidative stress treatment *in vitro* (Mcmahon et al., 2001). Furthermore, HpL3-4 cells expressing PrP(Δ110-119) lacking the α cleavage site were more protected against stress than those expressing an equivalent level of wild-type PrP (Mange et al., 2004). These observations suggest that PrP(Δ110-119) is the dominant positive form of PrP. Because the absence of α cleavage was found to enhance cell viability, α cleavage appears to suppress the protective activity of PrP. By contrast, β cleavage may contribute to protection. Further studies are required to analyze the effect of β cleavage on the neuroprotection mechanism of PrP.

Regarding OR and HR, several potential interactors of PrP have been reported. Copper specifically binds the OR of PrP (Hornshaw et al., 1995; Brown et al., 1997; Viles et al., 1999; Kramer et al., 2001). STI1, binds with amino acid residues 113-128 located in the N-terminal half of the HR of PrP (Zanata et al., 2002). Indeed, immunoprecipitation studies suggest that STI1 interacts with PrP in HpL3-4-PrP cells (Sakudo et al., 2005a). Therefore, we performed additional studies using HpL3-4 cells in order to determine how STI1 and copper might contribute to PrP-dependent anti-oxidative signaling. To investigate whether the STI1 is important for the biological activities displayed by PrP, the effect of the inhibitory peptides against PrP-STI1 on HpL3-4-EM cells was compared to that on HpL3-4-PrP cells under serum-free conditions (Sakudo et al., 2005a). The inhibitory peptides are toxic to HpL3-4-PrP cells due to inhibiting the SOD activity, although this is not the case for HpL3-4-EM cells (Sakudo et al., 2005a). Regarding copper, the cellular copper concentration was decreased in HpL3-4-EM cells, but not in HpL3-4-PrP cells under serum deprivation (Sakudo et al., 2004). Therefore, it is proposed that STI1 and copper might be involved in PrP-dependent SOD activation that can inhibit apoptosis *via* the OR and N-terminal half of PrP.

By considering the data reported using HpL cell lines, it is further proposed that the susceptibility of HpL3-4 cells to cell death is probably due to reduced SOD activity, induced, at least in part, by the absence of PrP-STI1 signals and the abnormality of copper homeostasis as well as the lack of β cleavage. Taken together, these findings suggest that PrP plays an anti-oxidative role and functions only under extreme stress such as oxidative conditions.

## Conclusions and future perspectives

The list of abnormalities caused by PrP-deficiency from the results of phenotype analysis of *Prnp*^−/−^ mice and from comparison between PrP-non-expressing cells and PrP-expressing cells continues to grow. Nonetheless, there has been little consensus from these studies regarding the overall main cellular function of PrP^C^. To enable us to analyse PrP^C^ in detail, *Prnp*^−/−^ cell lines have been established. Recently, the number of available *Prnp*^−/−^ cell lines has increased, including neuronal cells, astroglial cells, microglia, macrophages, and fibroblasts (Table 1). Rescue of *Prnp* by reintroducing into *Prnp*^−/−^ cell lines will provide convincing data. If further evidence is required, genetic complementation using the PrP transgene should be performed using a vector driven *Prnp* promoter. Indeed, PrP^C^ function may differ depending on its expression level. As a consequence, levels of PrP^C^ expression that are either too high or too low may mask the true biological function of the protein. Indeed, overexpression may cause the emergence of different functions from the real physiological function. Thus, if possible, analysis should be performed using physiological expression levels of PrP^C^ in each cell type. However, in the case of low PrP^C^ expressing cells, it becomes very difficult to detect the PrP^C^ expression and analyse PrP^C^ function (Raeber et al., 1999; Prinz et al., 2004).

**Table 1:** ***Prnp*^−/−^ cell lines and their characteristics**.

**Names**	**Method of production of cell lines**	**Cell type**	***Prnp*^−/−^ mouse origin**	**Culture medium**	**Main characterization compared to PrP expressing cells**	**References**
HpL2-1, HpL3-2, HpL4-3	Retrovirus-mediated method by SV40 large T antigen expressing vector	Neuronal-precursor cells (expessing NF- 68K)	Rikn	10% FCS-DMEM	Susceptible to serum deprivation	Kuwahara et al., 1999
Zpl2-1, Zpl2-4, Zpl3-4	Lipofection of SV40 large T antigen expressing vector	Neuronal cells (expressing NeuN)	ZrchI	DMEM	Higher prolifertion rate	Kim et al., 2005
SFK-B, SFK-C	Continuous culture of abdominal skin explants	Skin fibroblast cells	Ngsk	10% FCS-DMEM	Decreased expression of Ras and Rac related proteins	Satoh et al., 1998, 2000
F14	Fusion of PrP-knockout cerebellar cells and mouse neuroblastoma cells	Neuronal cells (expressing NeuN, MAP-2, and synaptophysin)	Npu	Serum containing DMEM	Similar distribution of GFP-PrP fusion protein	Holme et al., 2003
NpL2	Retrovirus-mediated method by SV40 large T antigen expressing vector	Neuronal cells (expressing NF-L, NF-M, NF-H, and MAP-2)	ZrchI	10% FCS-NB/B27 medium	Susceptible to serum deprivation	Nishimura et al., 2007
MG0	Retrovirus-mediated method by c-myc expressing vector	Microglial cell (expressing Mac-1 and F4/80)	Rikn	10% FCS-DMEM supplemented with 100 μM β-mercaptoethanol, 10 μg/ml insulin	Comparison has not been performed	Iwamaru et al., 2007
MpLZ4-3	Retrovirus-mediated method by SV40 large T antigen expressing vector	Macrophage (expressing MOMA-2 and F4/80)	ZrchI	10% FCS-DMEM	Shorter pseudopodium extension and less phagocytotic activity	Uraki et al., 2010
PrP^0/0^/1	Treatment with chemical mutagen (3-methylcholanthrene)	Embryonic fibroblast (expressing fibronectin)	Ngsk	10% FCS-MEMD supplemented with 2 mM-glutamine	Higher prolifertion rate (probably artificial)	Prcina et al., 2010
GpL1	Retrovirus-mediated method by SV40 large T antigen expressing vector	Glial cells (expressing GFAP)	ZrchI	10% FCS-DMEM	Susceptible to serum deprivation	Nishimura et al., 2008

*DMEM, Dulbecco's modified Eagle's medium*.

*MCA, Methylcholanthrene, which is a highly carcinogenic polycyclic aromatic hydrocarbon produced by burning organic compounds at very high temperatures*.

*NB/B27 medium, neurobasal medium (NB) (Gibco BRL, Gaithersburg, MD, USA) supplemented with B27 supplement (B27) (Gibco) and glutamine*.

*Updated from Table 3 in Sakudo et al. (2006) with permission from Bentham Science Publishers, Ltd*.

The use of the *Prnp*^−/−^ cell lines has facilitated the identification of abnormalities caused by PrP-deficiency at the cellular level (Table 2). Establishment of more *Prnp*^−/−^ cell lines will further contribute to our understanding of PrP^C^ function. For example, PrP family members are known to be expressed in reproductive tissues, such as testis, ovary, and placenta (Bonnet and Pailhoux, 2014; Makzhami et al., 2014). Thus, cells derived from reproductive tissues should also be used for the establishment of *Prnp*^−/−^ cell lines. The importance of PrP^C^ in leukocyte function has also been suggested because *Prnp*^−/−^ lymphocytes show a reduction of mitogen response, cytokine production and proliferation (Kubosaki et al., 2003; Bainbridge and Walker, 2005). Recent studies have also demonstrated that PrP^C^ plays a role in T cell and dendritic cell interactions (Ballerini et al., 2006). Therefore, the establishment of a *Prnp*^−/−^ leukocyte cell line would be interesting.

**Table 2:** **Abnormality of PrP gene-deficient cell lines**.

**Names**	**Main characterization compared to PrP expressing cells**	**References**
HpL2-1, HpL3-2, HpL4-3	Higher rate of apoptosis by serum deprivation	Kuwahara et al., 1999; Sakudo et al., 2003a, 2005a,b,c; Kim et al., 2004; Vassallo et al., 2005; Wu et al., 2008
HpL3-4	Decrease of intracellular copper concentration after serum deprivation	Sakudo et al., 2004
HpL3-4	Decrease of cellular SOD activity	Sakudo et al., 2003a
HpL3-4	Bigger increase of intracellular superoxide anion after serum deprivation	Sakudo et al., 2003a
HpL3-4	Reduced increase of intracellular hydrogen peroxide after serum deprivation	Sakudo et al., 2003a
HpL3-4	Bigger increase of caspase-3/9 activation after serum deprivation	Sakudo et al., 2003a
HpL3-4	Increased sensitivity of poliovirus infection	Baj et al., 2005
HpL3-4	Increased coxsackievirus B3 production and apoptotic cell death	Nakamura et al., 2003a
HpL3-4	Alteration of Ca^2+^ concentration in mitochondria after serum deprivation	Kim et al., 2004
HpL3-4	Alteration of transmembrane potentials in mitochondria after serum deprivation	Kim et al., 2004
HpL3-4	Alteration of cytochrome c level in mitochondria after serum deprivation	Kim et al., 2004
HpL3-4	Higher rate of apoptosis by SIN-1	Vassallo et al., 2005
HpL3-4	Reduced PI 3-kinase activity	Vassallo et al., 2005
HpL3-4	Decrease of cellular viability	Mange et al., 2004; Christensen and Harris, 2008
HpL3-4	Shorter neurite extension after differentiation	Kuwahara et al., 1999
HpL3-4	Suceptible to amyloid beta toxicity and amyloid beta-inducing autophagy	Nah et al., 2013
HpL3-4	Decreased rise in intracellular calcium following hydrogen peroxide treatment	Krebs et al., 2007
HpL3-4	Neutralization of Dpl toxicity	Sakudo et al., 2005c
NpL2	Decrease of SOD activity	Nishimura et al., 2007
NpL2	Higher rate of apoptosis by serum deprivation	Nishimura et al., 2007
Zpl2-1, Zpl2-4, Zpl3-4	Higher prolifertion rate	Kim et al., 2005
Zpl3-4	Increased autophagy induced by serum deprivation	Oh et al., 2008
Zpl3-4	Higher rate of apoptosis and autophagy by serum deprivation	Oh et al., 2008
GpL1	Decrease of SOD activity	Nishimura et al., 2008
GpL1	Higher rate of apoptosis by serum deprivation	Nishimura et al., 2008
GpL1	Decrease of cellular viability of co-cultured neuronal cells	Onodera and Sakudo, unpublished results
MplZ3-4	Shorter pseudopedium extension and reduced phagocytotic activity	Uraki et al., 2010
PrP^0/0^/1	Higher proliferation rate (may be non-specific)	Prcina et al., 2010
SFK-B, SFK-C	Decreased expression of Ras and Rac related proteins	Satoh et al., 1998, 2000

*PI3, phosphatidylinositol 3*.

*SIN-1, 3-morpholinosydnonimine*.

*SOD, superoxide dismutase*.

*Updated from Table 4 in Sakudo et al. (2006) with permission from Bentham Science Publishers, Ltd*.

As knockout animals lack all PrP expression, it is unclear whether the alteration and phenotypes in *Prnp*^−/−^ mice are attributable to defects in cell function or development. Function of PrP^C^ in the maturation process is feasible (Martin-Lannerée et al., 2014). Recent developments in regenerative medicine enable us to use various stem cells, such as embryonic stem (ES) cells and induced pluripotent stem (iPS) cells (Yamanaka, 2007). Establishment of *Prnp*^−/−^ ES or iPS cells would open the opportunity for detailed analysis of PrP^C^ function in reprogramming, expansion, isolation and differentiation.

The most studied phenotype of *Prnp*^−/−^ mice is myelin degeneration. This phenotype is seen in both type 1 and type 2 *Prnp*^−/−^ mice (Nishida et al., 1999; Baumann et al., 2007; Bremer et al., 2010). The myelin degeneration phenotype is caused by a deficiency of PrP^C^, suggesting that myelin maintenance may be a representative physiological function of PrP^C^. The process of demyelination is usually related to an abnormality of the immune system (Ramesh et al., 2013). The abnormal activity of immune cells may cause demyelination. Indeed, defects of immune cells in *Prnp*^−/−^ mice have been reported in several papers. Myelin sheath is composed of Schwann cells and oligodendrocytes (Alberts et al., 2008). As Schwann cell and oligodendroglia is one of glial cells, the study using glial *Prnp*^−/−^ cell line GpL1might reveal some characteristics of PrP^C^ related to myelin maintenance.

**Figure 4:**
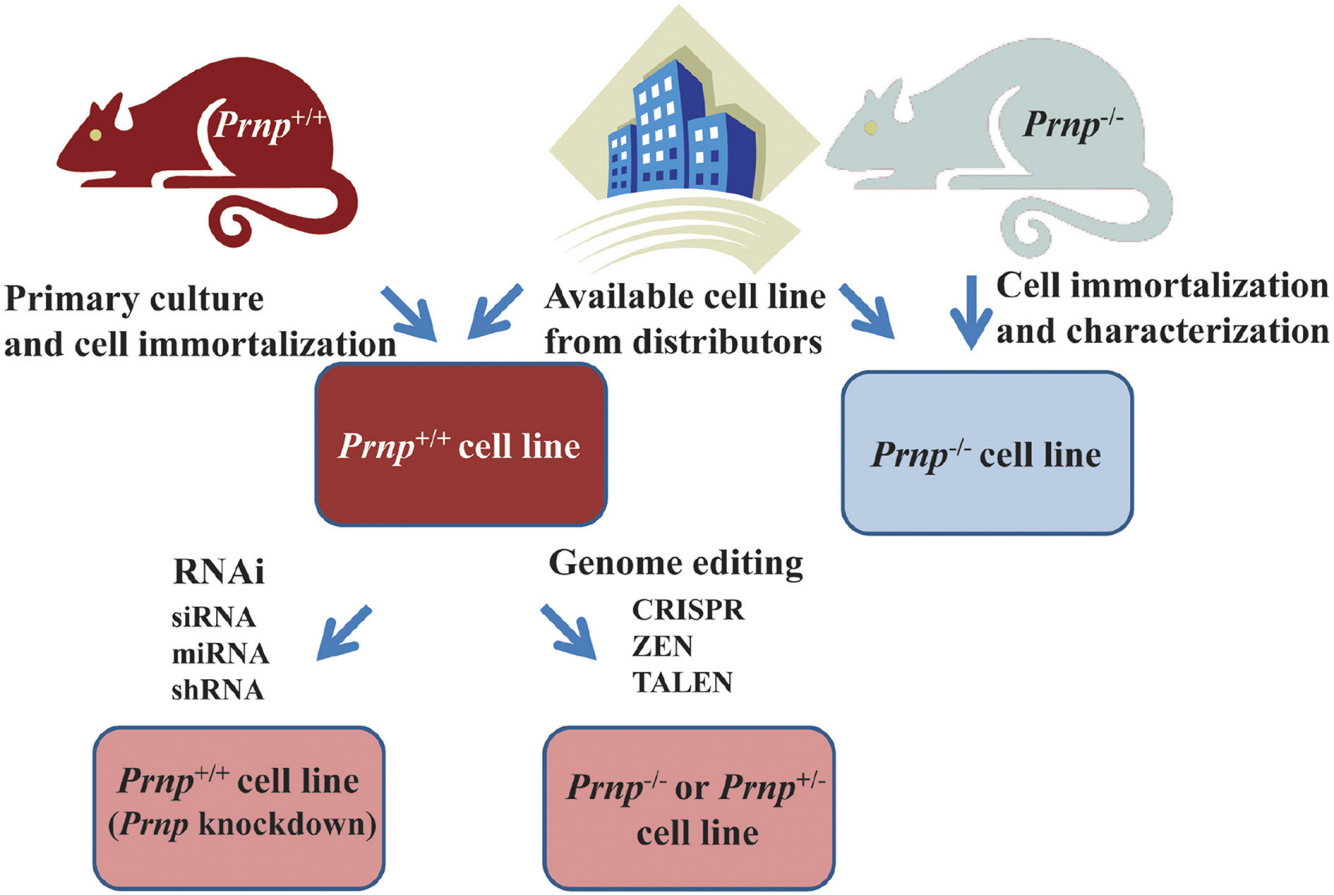
**Strategies for obtaining cell lines in which *Prnp* is suppressed or knocked out**. Established *Prnp*^−/−^ cell lines can be obtained from a number of distributors. If suppression of *Prnp* is required, rather than a knockout of *Prnp*, gene silencing technology using siRNA (small interference RNA), miRNA (micro-RNA) bi-shRNA (bifunctional short hairpin RNA) or shRNA (short hairpin RNA) is applicable. In other cases, when a new *Prnp*^−/−^ cell line is generated, immortalization of cells derived from *Prnp*^−/−^ mice is usually performed. Alternatively, gene editing techniques such as CRISPR (Clustered Regularly Interspaced Short Palindromic Repeat), ZFN (Zinc Finger Nuclease), or TALEN (Transcription activator-like effector nuclease) can be used to knockout *Prnp*, resulting in the production of *Prnp*^+/−^ or *Prnp*^−/−^ cell lines. Cells can be immortalized using oncogene transfection, radiation, fusion with cancer cells, or treatment with chemical mutagens. In other cases, cells derived from transgenic mice with an oncogene (e.g., temperature sensitive SV40 (Simian virus 40) large T, p53 etc.) that is overexpressed, mutated or deleted can proliferate and be maintained in culture medium. Continuous fibroblast culture can also be used. A cell line may be established after cloning and selection of these cells.

If knockout of *Prnp* is not required, suppression of *Prnp* is relatively easily achieved by gene silencing technology using siRNA (small interference RNA), miRNA (micro-RNA), bi-shRNA (bifunctional short hairpin RNA), or shRNA (Kubowicz et al., 2013). Indeed, shRNA of *Prnp* has been used for producing PrP^C^-depleted 1C11 neuroectodermal cells (Loubet et al., 2012). If a knockout of *Prnp* is required, the relevant *Prnp*^−/−^ cell line should be generated (Figure 4). Recent development of immortalization techniques enables cells derived from *Prnp*^−/−^ mice to be immortalized using oncogene transfection, radiation, fusion with cancer cells or treatment with chemical mutagens. In other cases, cells derived from transgenic mice with an oncogene (e.g., temperature sensitive SV40 largeT or p53) that is overexpressed, mutated or deleted can proliferate and be maintained in culture medium, resulting in the establishment of a cell line (Obinata, 2007). Alternatively, gene editing techniques such as CRISPR (Clustered Regularly Interspaced Short Palindromic Repeat) (Hsu et al., 2014), ZFN (Zinc Finger Nuclease) (Swarthout et al., 2011) or TALEN (Transcription activator-like effector nuclease) (Wright et al., 2014) can be used to knockout *Prnp*, resulting in the production of *Prnp*^+/−^ or *Prnp*^−/−^ cell lines.

*Prnp*^−/−^ cell lines are mainly used for two experimental purposes; analysis of PrP^C^ function and the study of prions. For example, there are several reports of the successful production of monoclonal and polyclonal antibodies against PrP by immunizing *Prnp*^−/−^ mice with recombinant murine PrP (Zanusso et al., 1998), human recombinant PrP folded into α or β (Beringue et al., 2003), purified PrP^C^ and PrP^Sc^ (Prusiner et al., 1993; Tayebi et al., 2011), scrapie-infected mouse neuroblastoma cells (Nakamura et al., 2003a), DNA plasmids encoding *Prnp* (Krasemann et al., 1996a,b), PrP-displaying retrovirus particles (Nikles et al., 2005), and PrP-coated microbeads (Tayebi et al., 2011). Thus, by immunizing *Prnp*^−/−^ mice with the derived *Prnp*^−/−^ cell lines transfected with *Prnp* or mutated *Prnp*, specific antibodies to wild-type or mutated PrP could be readily obtained. Furthermore, to obtain specific antibodies to PrP^Sc^, immunization of *Prnp*^−/−^ mice with prion-infected *Prnp*^−/−^ cells transfected with *Prnp* could be a promising approach.

There is a species barrier in prion susceptibility, which is determined by the difference between the species of the host expressing PrP and species of prion agent (Beringue et al., 2008). *Prnp*^−/−^ cell lines are useful for producing susceptible cells to target species of prion agent after *Prnp* transfection of the target species. Importantly, endogenous PrP is not present in the *Prnp*^−/−^ cell line. Thus, interference by endogenous PrP does not occur in a *Prnp*^−/−^ cell line. The *Prnp*^−/−^ cell line transfected with deletion mutants of PrP can be used for investigating important regions of PrP for prion infection. Previous results using the HpL3-4 cell line and chandler scrapie strain showed that OR is an essential region for the production of PrP^Sc^ during the early stage of infection (Sakudo et al., 2008). This was supported by other reports using the HpL3-4 cell line and 22L scrapie strain, indicating that substitution of amino acid residues of mouse PrP at position 96, located in the region between OR and HR, decreased PrP^Sc^ formation (Maas et al., 2007). The report also showed that the C-terminal half of PrP impacts on conversion efficiency. The C-terminal half of PrP includes regions forming a secondary structure of α-helix, termed α1-α3, while that of β-sheet is termed β 1 and β 2. Substitution of amino acid residues of mouse PrP at position 132 (located between β 1 and α1), 150 (α1 region), 167 (located between β 2 and α2), 189 (α2 region) and 204 (α3 region) abolished the efficient conversion of PrP^Sc^. Taken together, these results suggest OR, α-helices and the surrounding region contributes to the susceptibility of prion infection. Newly generated PrP^C^ and PrP^Sc^ antibodies together with cell lines that are susceptible or non-susceptible to target species of prion agents will be extremely useful for exploring prion biology.

Finally, we would like to emphasize that development of “OMICS” such as genomics, epigenomics, proteomics, metabolomics and transcriptomics will enable us to obtain a global view of the network of processes that are regulated by PrP^C^. By exploiting the combination of *Prnp*^−/−^ cell lines, comparison of PrP-expressing and PrP-non-expressing cells by OMICS will provide invaluable information on the effect of PrP-deficiency and fundamental differences caused by PrP-deficiency. The accumulated knowledge using these systems will be of great help in understanding PrP^C^ functions, which is also important in terms of clarifying the etiology of prion diseases.

## Author contributions

All authors contributed to the conception of this review and approved the submitted version.

### Conflict of interest statement

The authors declare that the research was conducted in the absence of any commercial or financial relationships that could be construed as a potential conflict of interest.
